# Organ Transplantation: Current Status, Challenges, and Future Prospects

**DOI:** 10.1002/mco2.70567

**Published:** 2026-01-08

**Authors:** Xinqiang Li, Ruidong Ding, Jinzhen Cai

**Affiliations:** ^1^ Organ Transplantation Center Affiliated Hospital of Qingdao University Qingdao China; ^2^ Institute of Organ Donation and Transplantation Medical College of Qingdao University Qingdao China

**Keywords:** immune engineering, organ regeneration, organ transplantation, transplant immunology, xenotransplantation

## Abstract

Organ transplantation has progressed from a life‐saving surgical intervention to a multidisciplinary field integrating immunology, bioengineering, and data science. Despite major advances in donor management, perioperative care, and immunosuppression, long‐term graft survival remains limited due to chronic rejection, infection, and organ scarcity. Recent breakthroughs in organ preservation, particularly in hypothermic and normothermic machine perfusion, have enabled real‐time graft assessment, metabolic reconditioning, and localized therapeutic delivery. Emerging precision immunomodulation strategies, including regulatory T‐cell therapy, gene‐edited cellular platforms, tolerogenic dendritic cells, and biomarker‐guided minimization, are reshaping alloimmune control toward durable tolerance. Innovations in xenotransplantation, multigene‐edited donor animals, and tissue biofabrication offer potential solutions to structural organ shortages, although they are accompanied by regulatory and ethical challenges. Artificial intelligence further enhances donor–recipient matching, risk prediction, and personalized immunosuppressive management. This review synthesizes advances in preservation technologies, immune engineering, cellular tolerance induction, artificial intelligence‐driven decision support, and xenotransplantation and provides a comprehensive overview of the evolving transplant landscape. By integrating mechanistic insights into translational progress, we outline future pathways for regenerative, immune‐educational, and precision organ medicine.

## Graphical abstract

Organ transplantation success relies on precise modulation of the alloimmune response across distinct temporal phases of rejection. Hyperacute rejection results from preformed antibodies activating complement and causing thrombosis, while acute rejection involves T cell‐ and antibody‐mediated mechanisms that drive endothelial activation and microvascular inflammation. Chronic rejection reflects maladaptive repair and fibroproliferation leading to progressive graft dysfunction. Contemporary management integrates induction and maintenance immunosuppression with advanced molecular monitoring. Balancing immune control against infection, toxicity, and malignancy remains central to optimizing long‐term graft survival. Together, these advances signify a paradigm shift from empirical suppression toward precision‐guided, immune‐educational transplant medicine.

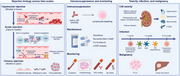

## 1 Introduction

Organ transplantation has evolved from an experimental surgery to a routine life‐saving therapy for end‐stage organ failure. In the earliest era of transplantation, only highly selected patients and centers were able to attempt homotransplantation. Over the past several decades, advances in donor evaluation and management, surgical techniques, graft preservation, postoperative critical care, and immunosuppression have driven substantial improvements in early graft survival and patient outcomes [1, 2]. For instance, improvements in surgical anastomotic techniques, better perioperative hemodynamic management, refined donor selection, expanded criteria donors, and incremental gains in immunosuppressive regimens have enabled transplantation to become the standard of care for many end‐stage organ failures.

Nevertheless, the supply of organs remains far short of demand and long‐term graft attrition persists. Although early posttransplant survival rates have improved steadily, the lifetime benefits that a recipient can derive remain constrained by chronic graft injury, late immunological and nonimmunological insults, infection, malignancy, and comorbidities. The underlying issue reflects intertwined innate and adaptive alloimmune pathways, as well as ischemia–reperfusion injury (IRI), donor organ quality decline, and recipient‐related factors [3, 4]. Despite improvements to tailored drug combinations and emerging biomarkers for monitoring allograft status, chronic dysfunction continues to limit the long‐term benefits.

Recently, new preservation strategies have extended graft viability, enabling the objective assessment of marginal organs and opening a therapeutic window to rehabilitate previously discarded grafts [5, 6, 7]. Currently, the field is shifting from broad immunosuppression to more precise, protolerogenic strategies and design methods to expand the donor pool through innovation, including xenotransplantation and bioengineering. Cellular therapies, gene editing, vector‐based modulation, and ex vivo biological delivery during machine perfusion are all directed to attenuate IRI and reprogram local immunity in the graft microenvironment [4, 8]. Genetically engineered porcine organs are advancing xenotransplantation as a potential solution to structural shortages while raising distinct considerations regarding safety, monitoring, ethics, and policy [8, 9].

This review synthesizes current practices and outcomes, appraises the evidentiary status of novel cellular and gene‐edited strategies, and evaluates the translational horizon of xenotransplantation, highlighting pragmatic paths to bring these innovations into routine care. While transplantation has matured into an established clinical modality, the next decade is poised to test whether the field can shift from “transplantation” to “regeneration,” from “suppression” to “re‐education” of immunity, and from scarcity to scalability.

## 2 Current Clinical Landscape

The global landscape of organ transplantation has entered an era of quantitative expansion and qualitative diversification, characterized by the increasing adoption of advanced preservation technologies, precision immunomodulation, and international standardization of data reporting. Activity and outcomes vary substantially across regions; however, common challenges persist, particularly organ scarcity, chronic graft attrition, and long‐term immunosuppressive complications.

### 2.1 China: Rapid Development and Structural Expansion

Over the past decade, China's organ donation and transplantation systems have undergone remarkable expansion and restructuring in recent years. According to Shi et al., the number of deceased‐donor organs per million population (pmp) rose from 0.03 pmp in 2010 to 3.69 pmp by 2018, and the number of transplant surgeries, including kidney, liver, heart, and lung, increased substantially in the same period [10]. The comprehensive reform of the donation system marked a pivotal turning point: on January 1, 2015, China formally adopted mandatory voluntary citizen donation as the only legitimate source for transplantation, ending prior reliance on organs from executed prisoners and establishing the “China mode” of donation and transplant governance [11]. During this reform, China also built a nationwide infrastructure, including the China Organ Transplantation Development Foundation, China National Human Organ Donation and Transplantation Committee, and China Organ Transplant Response System for the transparent allocation and sharing of organs. Collectively, these advances underscore the country's transition from quantity expansion to quality refinement in both surgical practice and regulatory governance.

### 2.2 United States: Mature Registry and Outcome Stability

Across high‐income systems, contemporary transplant activity and outcomes are best characterized by the OPTN/SRTR peer‐reviewed Annual Data Reports and organ‐specific chapters in the American Journal of Transplantation. For the most recent fully published cycle, the kidney chapter documents a record of 28,142 transplants, largely driven by growth in deceased‐donor procedures, improved utilization of marginal kidneys, and expansion of paired‐exchange networks [12]. The 2023 liver report similarly recorded 10,659 transplants, attributing gains to increased recovery from older age, donation after circulatory death (DCD) donors and wider availability of hypothermic oxygenated machine perfusion (HOPE) [13]. Heart‐transplant epidemiology shows historically low adult wait‐list mortality (<5 %) alongside stable volumes [14]. Lung transplant programs report increased access and shorter time‐to‐transplant for most candidates, aided by ex vivo lung perfusion (EVLP) and broader donor sharing. One‐year posttransplant survival now exceeds 94% for kidney and 92 % for liver recipients, yet chronic immune‐mediated injury and metabolic comorbidities continue to erode long‐term graft longevity. Chronic phenotypes, including cardiac allograft vasculopathy (CAV) and chronic lung allograft dysfunction (CLAD), remain dominant late complications [15].

### 2.3 Global Epidemiology and Postpandemic Recovery

Outside the United States and China, global patterns have been synthesized through the World Health Organization‐affiliated Global Observatory on Donation and Transplantation, which compiles standardized data from >90 countries. Between 2010 and 2019, worldwide solid‐organ transplant activity increased by nearly 40%, led by Western Europe, Asia‐Pacific regions, and Latin America. The COVID‐19 pandemic caused a 20–30 % decline in activity during 2020, followed by a recovery, to prepandemic levels by 2022 [16]. Nonetheless, marked disparities remain: Spain, the United States, and France achieve >40 donations pmp, whereas rates in many middle‐income nations remain at <5 pmp. Legal frameworks, public awareness, and logistics infrastructure continue to define regional heterogeneity [17].

Collectively, China's rapid institutional growth, the United States’ mature registry‐based optimization, and global postpandemic recovery illustrate the multilevel maturation of transplantation medicine. Short‐term survival now approaches physiological limits; however, yet durable graft longevity, equitable access, and cost‐effective precision monitoring remain the defining frontiers for the coming decade.

## 3 Immunological Barriers and Present‐Day Management

**FIGURE 1:**
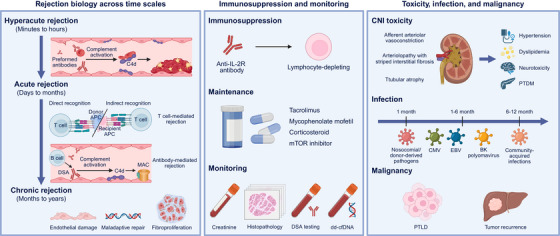
Immunological challenges in organ transplantation and present‐day management strategies. Left panel: Rejection biology across time. Hyperacute rejection, occurring within minutes to hours, is mediated by preformed antibodies that activate complement and cause thrombosis. Acute rejection, which occurs from days to months, involves T cell‐mediated rejection (TCMR) and antibody‐mediated rejection (AMR). TCMR arises through distinct allorecognition pathways, direct and indirect, leading to endothelial activation and microvascular injury. Chronic rejection, over months to years, results in persistent endothelial damage, maladaptive repair, and fibroproliferation, with organ‐specific manifestations. Middle panel: Immunosuppression and monitoring; illustrates the immunosuppressive treatments, such as anti‐IL‐2R antibodies for induction and calcineurin inhibitors, mycophenolate mofetil, and mTOR inhibitors for maintenance. It also highlights the monitoring strategies, including creatinine, histopathology, DSA testing, and dd‐cfDNA, to assess graft function and rejection risk. Right panel: Toxicity, infection, and malignancy; depicts the risks of immunosuppression, such as CNI nephrotoxicity, infection timelines, and malignancy concerns like PTLD and tumor recurrence. This figure was created with BioRender (https://biorender.com/).

Transplantation success hinges on the precise control of the alloimmune response, the host's recognition of non‐self‐histocompatibility antigens expressed by the graft. Despite the substantial progress in surgical techniques and immunosuppression, rejection remains the principal barrier to durable tolerance and long‐term survival. The immunologic dialogue between the donor and recipient evolves over time, encompassing hyperacute, acute, and chronic phases that reflect distinct cellular and molecular mechanisms (Figure 1). Understanding the temporal dimensions of rejection is essential for designing targeted interventions, refining biomarker‐based monitoring, and guiding rational immunosuppression minimization.

### 3.1 Rejection Biology Across Time

Hyperacute rejection (HAR) occurs within minutes to hours when preformed antibodies, most commonly anti‐ABO, anti‐HLA, and some non‐HLA specificities, bind to the graft endothelium, activate the complement with C4d deposition, and precipitate platelet‐rich thrombosis. Rigorous cross‐matching and desensitization have made such events uncommon, but they remain a concern for highly sensitized candidates [18, 19, 20].

Acute rejection, comprising T‐cell‐mediated rejection (TCMR) and antibody‐mediated rejection (AMR), emerges over several days to months. TCMR is initiated through three cooperative allorecognition pathways: direct recognition of intact donor MHC on donor APCs, indirect recognition of processed donor peptides on recipient MHC, and the now well‐substantiated semi‐direct pathway, in which recipient APCs acquire intact donor MHC and present them to host T cells. These pathways drive endothelial activation, microvascular inflammation, and interstitial/vascular injury, even under calcineurin‐inhibitor‐anchored regimens, explaining why current maintenance reduces, but does not abolish, TCMR [21, 22]. AMR is initiated when donor‐specific antibodies (DSAs) engage graft microcirculation, activating complement and Fc‐dependent effector functions. Complement‐binding C1q‐positive DSA identifies recipients at heightened risk of AMR and graft loss versus C1q‐negative DSA, supporting their use for risk stratification and biopsy triage [23, 24, 25]. Pathologically, AMR encompasses active, chronic active, and chronic categories in the Banff classification, integrating microvascular injury, C4d, serology, and molecular injury scores; however, C4d‐negative AMR is recognized, underscoring the importance of multimodal assessment [26, 27].

Chronic rejection evolves over months to years and shows organ‐specific phenotypes driven by persistent endothelial damage, maladaptive repair, and fibroproliferation: Liver—chronic rejection in liver transplantation is defined by progressive loss of interlobular bile ducts, obliterative arteriopathy, and vanishing bile‐duct syndrome caused by persistent alloimmune injury to the biliary epithelium and endothelium [28]. Kidney transplant glomerulopathy is the histological hallmark of chronic active AMR and strongly predicts progressive loss of function. Management remains largely based on consensus due to limited, high‐grade trial data [29, 30]. Heart—CAV manifests as diffuse concentric intimal hyperplasia of the epicardial and intramyocardial arteries, often with microvascular dysfunction, which remains a leading late cause of graft loss despite modern care [31, 32, 33]. Lung—CLAD comprises BOS, RAS, mixed, and undefined phenotypes; CLAD dominates long‐term mortality after lung transplantation, and is influenced by alloimmunity, infection, and aspiration, among others [34, 35].

### 3.2 Present‐Day Immunosuppression and Monitoring

Contemporary immunosuppression generally begins with an induction phase, typically using an anti‐IL‐2‐receptor antibody (such as basiliximab) or a lymphocyte‐depleting agent (such as antithymocyte globulin or alemtuzumab), followed by a maintenance phase anchored by a calcineurin inhibitor (CNI), such as tacrolimus combined with mycophenolate mofetil. Many centers use early corticosteroid withdrawal or minimization in low‐immunological‐risk recipients, recognizing that long‐term steroid exposure drives metabolic, cardiovascular, and bone complications. When CNI toxicities (nephrotoxicity, hypertension, and dyslipidemia) or other constraints (donor age and recipient comorbidity) arise, some centers move toward belatacept‐based costimulation blockade or mTOR inhibitor‐based strategies (such as everolimus), either de novo or via conversion [36]. For instance, in kidney transplantation, multiple long‐term studies have shown that belatacept yields superior renal function and graft/patient survival compared with cyclosporine or tacrolimus [37, 38]. Registry and conversion cohort data further support the role of belatacept in minimization strategies, although its early rejection rate may be higher in selected phenotypes [39, 40].

Modern transplantation monitoring has evolved from creatinine/trough levels and biopsy alone to integrate histopathology, DSA testing, and molecular surveillance tools. Among these, donor‐derived cell‐free DNA (dd‐cfDNA) has emerged as a noninvasive biomarker capable of identifying graft injury earlier than conventional markers [41]. A recent prospective study of 106 kidney transplant recipients found that dd‐cfDNA levels at the time of biopsy strongly correlated with histologic rejection [42]. Another surveillance study reported that dd‐cfDNA levels >0.5% at 2 months posttransplantation were associated with worse estimated glomerular filtration rate at 1 year [43]. Despite incremental progress, the treatment of established AMR still largely relies on expert consensus, with limited high‐grade evidence [44, 45].

### 3.3 Toxicity, Infection, and Malignancy Under Immunosuppression

CNIs acutely induce dose‐dependent afferent arteriolar vasoconstriction and characteristic chronic arteriolopathy with striped interstitial fibrosis and tubular atrophy over time. Protocol histology series and contemporary reviews describe hyaline arteriolopathy, tubular atrophy, and “striped” fibrosis as the classic CNI injury pattern; although tacrolimus often yields fewer early rejection episodes than cyclosporine, both drugs are linked to similar chronic arteriolar lesions at long follow‐up. These renal changes coexist with systemic toxicities, including hypertension, dyslipidemia, neurotoxicity, and posttransplant diabetes mellitus, which motivate trough‐guided dose optimization and, in selected phenotypes, conversion to alternative regimens [46, 47, 48, 49].

Despite prophylaxis, infections peak in the first posttransplant year, following a fairly reproducible timeline: first month, nosocomial/surgical and donor‐derived pathogens; Months 1–6, opportunistic and immunomodulating viruses (notably CMV, EBV, and BK polyomavirus); and after 6–12 months, community‐acquired infections dominate, unless net immunosuppression remains high. Recent studies reaffirm the central roles of CMV and BKPyV in kidney recipients, both in direct morbidity and as amplifiers of alloimmunity, and note that mTOR‐based regimens may lower CMV/BK incidence in some contexts at the cost of other toxicities [50, 51, 52].

Cancer remains a major long‐term concern after organ transplantation, and its risk is closely linked to the cumulative intensity and duration of immunosuppression. Posttransplant lymphoproliferative disorder (PTLD) is a prototypical malignancy, particularly in EBV‐seronegative recipients of EBV‐positive grafts or those under profound T‐cell suppression. Reduction of immunosuppression is the first‐line therapy, followed by rituximab‐based regimens, as indicated [53]. In liver transplantation, the recurrence of pre‐existing HCC remains a leading cause of late mortality, influenced by tumor biology, microvascular invasion, and the degree of posttransplant immunosuppression [54, 55]. Excessive calcineurin‐inhibitor exposure and prolonged corticosteroid use have been associated with tumor progression through proangiogenic and mTOR pathway activation, whereas mTOR inhibitors, such as sirolimus and everolimus, exert antiproliferative effects, and may reduce recurrence risk [56, 57].

## 4 Inducing Immune Tolerance and Precision Immunomodulation

Efforts to achieve donor‐specific immune tolerance and refine precise immunomodulation have accelerated markedly in recent years, reshaping the conceptual and therapeutic landscape of transplantation. Moving beyond the constraints of conventional pharmacological immunosuppression, contemporary strategies have focused on selectively modulating alloimmune responses through targeted molecular blockade, adoptive cellular therapies, hematopoietic chimerism, and tolerogenic myeloid platforms. Parallel advances in immune monitoring biomarkers have provided an increasingly reliable framework for individualized drug minimization and structured withdrawal. Collectively, these emerging approaches outline a multidimensional roadmap in which immune activation, regulation, and surveillance can be precisely adjusted to promote durable graft acceptance while minimizing systemic toxicity.

### 4.1 Costimulation Blockade in Clinical Practice

Over the past 5 years, costimulation blockade has evolved from a niche innovation to the central pillar of immunosuppressive strategy refinement. Belatacept (CTLA4–Ig) remains the leading clinical representative, validated by extended outcomes from the BENEFIT (NCT00256750, completed) and BENEFIT‐EXT (NCT00114777, completed) trials, showing superior renal function and a lower incidence of chronic allograft injury compared with CNIs [4]. Beyond improved glomerular filtration, long‐term belatacept use results in favorable metabolic, cardiovascular, and neurocognitive profiles, reinforcing its role as a calcineurin‐sparing anchor in stable kidney recipients.

#### 4.1.1 Mechanistic Advances and Evolving Clinical Context

Recent mechanistic studies have confirmed that belatacept effectively interrupts the CD28‐B7 costimulatory pathway, dampening T‐cell activation while preserving regulatory T (Treg)‐cell homeostasis [58]. Despite these benefits, early acute rejection remains more common in recipients with high pretransplant T follicular helper (Tfh) and effector memory T‐cell frequencies. This has prompted a trend toward combination induction regimens using lymphocyte‐depleting agents or IL‐2R antagonists to better control early immune priming [59].

#### 4.1.2 Belatacept Across Organ Platforms

Although kidney transplantation remains the primary domain, the organ‐agnostic immunomodulatory potential of belatacept is under active evaluation in the liver, pancreas, and vascularized composite allotransplantation (VCA). Studies on pancreas‐islet transplantation have shown encouraging synergy between belatacept and sirolimus in prolonging islet survival without nephrotoxicity [60]. In liver transplantation, pilot programs have suggested that belatacept can reduce calcineurin toxicity and fibrosis progression while maintaining graft stability, although further long‐term data are needed. In VCA and xenotransplantation, belatacept combined with CD40 blockade markedly reduces microvascular inflammation and graft‐infiltrating lymphocyte density, resulting in longer graft survival in preclinical primate and porcine models [61]. Therefore, safety considerations are crucial. Because belatacept is restricted to EBV‐seropositive recipients owing to PTLD risk, careful pretreatment screening and virological monitoring are mandatory [36, 62, 63]. Nonetheless, in a real‐world series, the incidence of PTLD under vigilant surveillance remained low and was comparable to that under conventional regimens.

#### 4.1.3 Beyond CD28–B7: Next‐Generation Costimulatory Targets

A new generation of costimulation inhibitors targeting the CD40/CD154 and ICOS/ICOSL axes has emerged as a potent adjunct or alternative to CTLA4–Ig. These pathways modulate B‐cell help and antibody generation, and their blockade prevents both cellular and humoral rejection. CD154 blockade has re‐emerged with improved safety: a recent study showed that an anti‐CD154 antibody effectively controlled AMR in sensitized nonhuman primate kidney recipients without thromboembolic events [64]. CD40 monoclonal antibodies, such as bleselumab and iscalimab have demonstrated safety in Phase II kidney and liver transplantation trials by reducing DSA formation and chronic allograft vasculopathy. The ICOS blockade, which targets the inducible costimulator ligand pathway, has shown synergistic effects when paired with anti‐CD40 therapy in islet xenotransplant models, reducing DSA generation and promoting regulatory immune signatures [58].

### 4.2 Cell Therapy to Reprogram Alloimmunity

**FIGURE 2:**
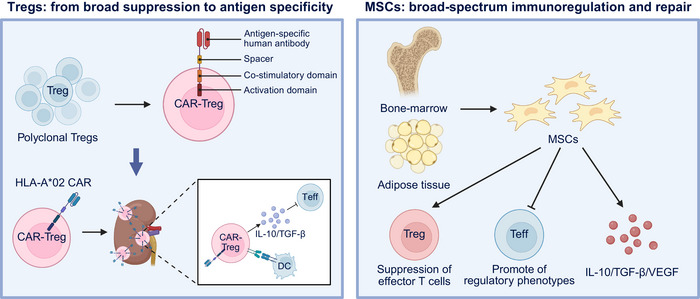
Regulatory T cells and mesenchymal stromal cells as complementary cell therapies for transplant tolerance. Left panel: Treg cells—from broad suppression to antigen specificity. Polyclonal Treg cells exert nonspecific immune suppression but show variable efficacy. Engineering with chimeric antigen receptors (CARs) enables donor antigen‐specific regulation, exemplified by HLA‐A*02‐targeted CAR–Treg cells. These cells localize to the graft, where they suppress effector T cells via IL‐10 and TGF‐β secretion and modulate dendritic cells, promoting local immune tolerance. Right panel: MSCs—broad‐spectrum immunoregulation and repair. Mesenchymal stromal cells (MSCs), derived from bone marrow or adipose tissue, mediate broad immunomodulatory and tissue‐repair functions. MSCs suppress effector T cells, promote regulatory phenotypes, and secrete IL‐10, TGF‐β, and VEGF to support tissue recovery and reduce inflammation. Together, Treg cells and MSCs represent synergistic approaches to retrain alloimmunity and achieve durable graft tolerance. This figure was created with BioRender (https://biorender.com/).

Instead of chronically suppressing the immune system using pharmacological agents, cell‐based immunotherapies seek to actively reeducate immune responses toward donor‐specific tolerance, thereby maintaining graft function while preserving systemic immunity. Two cellular platforms have emerged as the most advanced and complementary in this field: Treg cells, which enforce immune suppression through direct cell–cell contact and cytokine secretion, and mesenchymal stromal cells (MSCs), which exert broad immunomodulatory and tissue‐repair effects (Figure 2). Together, these modalities represent a paradigm shift from controlling rejection to retraining immunity, anchored in the principles of precision immunomodulation and personalized tolerance induction.

#### 4.2.1 Treg Cells: From Broad Suppression to Antigen Specificity

Cellular immunotherapy using Treg cells has moved from a concept to an early clinical reality. Initial studies employing polyclonal Treg cells expanded ex vivo and reinfused after transplantation have demonstrated safety and biological activity, but variable efficacy due to their broad, nonspecific repertoire. These limitations have driven the development of antigen‐specific Treg cell therapies, particularly chimeric antigen receptor‐Treg cells (CAR‐Treg cells) and TCR‐engineered Treg cells, which are designed to recognize donor antigens and deliver targeted immune suppression at the graft site.

The most clinically advanced example is TX200‐TR101, an autologous HLA‐A*02‐specific CAR‐Treg cell line developed for HLA‐A2‐mismatched kidney transplantation. Preclinical validation confirmed its stability, antigen‐driven activation, suppressive potency, and graft homing capacity [65]. The ongoing Phase 1/2 STEADFAST trial (NCT04817774, active) represents the first‐in‐human CAR‐Treg cell study assessing safety, persistence, and mechanistic efficacy, with an additional 15‐year follow‐up protocol (NCT05987527, enrolling) to monitor durability. Interim updates demonstrate successful manufacturing, with no major adverse events. Multiple trials within the ONE Study program and related consortia have evaluated the adoptive transfer of ex vivo‐expanded polyclonal Treg cells in kidney transplant recipients (NCT02088931, completed; NCT02145325, completed), showing acceptable safety and encouraging immunological modulation [66, 67].

Mechanistic studies indicate that CAR‐Treg cells localize preferentially to graft tissue, where they suppress effector T cells, regulate antigen‐presenting cells, and secrete IL‐10 and TGF‐β to dampen inflammation [68]. Despite this promise, key challenges persist, including ensuring long‐term lineage stability, defining optimal dosing and infusion timing, maintaining persistence in inflammatory milieus, and integrating therapy with CNIs, which may compromise Treg cell survival. The complexity and cost of autologous manufacturing also hinder scalability, motivating research on allogeneic or universal Treg cell platforms with genome editing to minimize host rejection [69].

#### 4.2.2 MSCs: Broad‐Spectrum Immunoregulation and Repair

Parallel progress has been made in MSCs therapy, which offers a versatile approach that combines immune regulation with tissue regeneration. MSCs modulate immune responses by suppressing effector T‐cells, promoting regulatory phenotypes, and releasing anti‐inflammatory cytokines and growth factors that enhance tissue repair. Early clinical studies on kidney and liver transplantation demonstrated their safety and biological activity. In a Phase I trial, autologous bone marrow MSCs (1 × 10^6^ cells/kg) reduced donor‐specific proliferation and improved subclinical rejection without adverse events, whereas liver transplant recipients receiving third‐party MSCs on postoperative Day 3 exhibited no increase in infection or malignancy, although they were not able to discontinue immunosuppression [70]. In liver transplantation, a prospective, controlled Phase I trial infused 1.5–3 × 10^6^/kg third‐party MSCs on postoperative Day 3 in 10 recipients under standard immunosuppression; no increase in infections or malignancy was observed in 12 months, although immunosuppression weaning was not successful [71].

Between 2021 and 2025, the field will expand beyond bone marrow sources. Adipose‐derived MSCs are highly immunomodulatory and are easier to scale [72]. New frontiers include MSC‐derived extracellular vesicles (EVs), which may replicate the immunoregulatory benefits of living cells while improving stability and storage logistics [73], and gene‐edited MSCs preconditioned for enhanced graft homing and survival [74]. Nonetheless, variability in the source, dose, and administration route continues to hinder reproducibility. MSC therapy currently serves as an adjunctive tool to reduce inflammation and support graft repair, rather than as a stand‐alone alternative to standard immunosuppression. The use of MSCs has evolved from rodent models to early clinical tests. Trials such as NCT01429038 (completed) and NCT02260375 (recruiting) investigated third‐party bone marrow‐derived MSCs administered around the time of liver or kidney transplantation, demonstrating feasibility and an acceptable safety profile, and suggesting a potential impact on rejection and biliary complications.

#### 4.2.3 Hybrid and Next‐Generation Cellular Strategies

The convergence of these cell types inspired a new generation of hybrid tolerance protocols. Researchers are exploring combinations of antigen‐specific Treg cells with MSCs or their EVs to achieve the synergistic suppression of alloimmunity while enhancing graft healing. Dual‐antigen or safety‐switch CAR Treg cells are being developed to increase precision and reversibility, and genome‐edited “off‐the‐shelf” universal Treg cells and MSCs are under investigation to simplify logistics and reduce costs. Emerging tools, such as biomarker‐guided dosing, cell tracking, and TCR‐clonotype sequencing, are being incorporated into early phase studies to optimize persistence and correlate immune signatures with clinical outcomes.

The integration of these therapies into mixed chimerism or tolerance‐induction protocols is gaining traction. A 2025 report from the International Society for Transplantation Immunology describes successful weaning from partial immunosuppression in small cohorts using early Treg cell enrichment combined with transient donor chimerism [75]. Such multidimensional designs exemplify a shift from single‐agent cell therapy to integrated immune‐reprogramming regimens.

### 4.3 Mixed‐Chimerism and Combined Organ–Marrow Strategies

**FIGURE 3:**
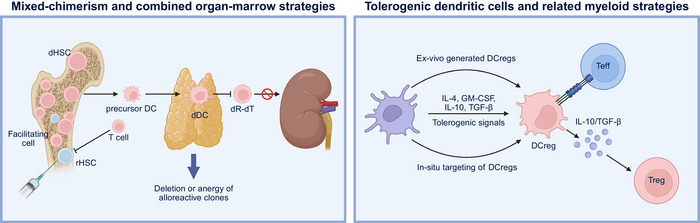
Mixed chimerism and tolerogenic dendritic cells strategies for transplant tolerance. Left panel: Mixed chimerism and combined organ‐marrow strategies. Establishing donor–recipient hematopoietic chimerism induces immune tolerance through deletion or anergy of alloreactive clones. Donor hematopoietic stem cells (dHSCs) and facilitating cells are infused with bone marrow under nonmyeloablative conditioning to generate donor‐derived dendritic cells (dDCs) in the thymus. These promote deletion of donor‐reactive T cells (dR‐dT), enabling durable or transient tolerance and long‐term graft survival. Right panel: Tolerogenic dendritic cells and related myeloid strategies. Dendritic cells can be conditioned ex vivo with tolerogenic cytokines (IL‐4, GM‐CSF, IL‐10, TGF‐β) or targeted in situ to induce regulatory phenotypes (DCregs). These DCregs suppress effector T cells and secrete IL‐10 and TGF‐β to promote regulatory T‐cell expansion, creating a tolerogenic immune environment and supporting graft acceptance. This figure was created with BioRender (https://biorender.com/).

Establishing donor–recipient hematopoietic chimerism can induce robust tolerance by deleting or anergizing alloreactive clones (Figure 3). Nonmyeloablative conditioning with perioperative donor bone marrow infusion produced mixed chimerism and long‐term renal allograft acceptance in nonhuman primates, and transitioned to clinical kidney transplantation, in which recipients achieved immunosuppression withdrawal under carefully controlled protocols [76, 77, 78]. Even transient chimerism can be sufficient for tolerance induction, with central deletional mechanisms persisting after donor hematopoietic cells disappear [79]. Transient versus durable chimerism may confer tolerance depending on the platform; however, the risks of infection, cytopenia, and graft‐versus‐host reactivity mandate meticulous patient selection and center expertise [80].

### 4.4 Tolerogenic Dendritic Cells (DCregs) and Related Myeloid Strategies

Autologous DCregs have reached Phase I/IIa testing in living‐donor kidney recipients, demonstrating their feasibility, good manufacturing practice (GMP)‐compliant manufacturing, and the capacity to taper adjunctive mycophenolate in selected patients without excess early rejection [81]. Donor‐derived regulatory DC infusion in liver transplantation has been demonstrated to modulate alloreactive immunity in vivo, reduce effector CD8+ T‐cell activation, and constrain natural killer (NK) cell cytotoxic signatures, consistent with enhanced peripheral regulation and a more permissive graft microenvironment [82]. A scalable route to deliver antigen‐presenting cells programmed to favor Treg cell induction and attenuate effector priming has been validated, while underscoring the need for standardized potency assays and longitudinal immune‐monitoring frameworks to confirm donor specificity and persistence (Figure 3).

### 4.5 Biomarker‐Driven Minimization and Withdrawal

While operational tolerance remains rare, it is biologically informative. Multicohort discovery has linked spontaneous kidney allograft tolerance to B‐cell‐enriched transcriptional signatures in blood and urine, and a consensus gene expression panel has been validated across datasets as a candidate tolerance biomarker set [83, 84]. In parallel, dd‐cfDNA has progressed from observational work to prospective clinical utility as a rejection activity signal that can guide biopsy and therapy. Recent reviews and translational studies support its integration into surveillance algorithms and its potential role in structured minimization trials, although definitive randomized evidence for safe withdrawal is still emerging [85, 86]. Building precision protocols will likely require composite decision rules that couple dd‐cfDNA with DSA kinetics, histopathology, or molecular microscopy scores and, where available, tolerance‐associated transcriptional signatures to safely reduce or discontinue drug exposure [83, 85, 87].

## 5 Technological Innovations Shaping the Field

Rapid advances in organ preservation, regenerative bioengineering, immune manipulation, and data‐driven medicine have reshaped the technological foundations of transplantation. As clinical demand continues to outpace donor supply and graft quality varies widely, emerging technologies aim to expand the usable organ pool, repair marginal grafts, engineer immunological compatibility, and optimize decision‐making throughout the transplant continuum. These innovations span the next‐generation ex vivo perfusion systems for dynamic preservation, metabolic repair, and biofabrication platforms that combine tissue engineering with 3D bioprinting to generate vascularized, personalized grafts. Complementing these are genetic and cellular engineering strategies that reshape immunogenicity and artificial intelligence (AI) tools that integrate multiomics and clinical data to guide prediction and precision care. Collectively, these converging fields delineate a new technological era in which preservation, repair, immune modulation, and analytics operate synergistically to improve graft viability, access, and long‐term transplant outcomes.

### 5.1 Next‐Generation Organ Preservation: Ex Vivo Perfusion Technologies

The persistent shortage of donor organs and increasing need to utilize marginal grafts have created major challenges for transplantation medicine. Conventional static cold storage (SCS), although long considered the gold standard, is limited in both preservation duration and the ability to assess organ function dynamically [88]. Because preservation quality directly affects graft viability and postoperative outcomes, improving organ maintenance methods is critical. In contrast to passive cooling of the SCS, ex vivo machine perfusion (EVMP) maintains metabolic activity and circulation outside the body, extending the safe preservation time while enabling real‐time assessment and active repair of the graft [89]. This dynamic platform has the potential to reduce complications and redefine graft selection, optimization, and allocation in clinical transplantation.

#### 5.1.1 Typology and Mechanistic Foundations of EVMP

EVMP systems can be classified based on the perfusion temperature and degree of physiological simulation. Normothermic machine perfusion (NMP) maintains a near‐physiological temperature using blood‐ or hemoglobin‐based perfusates, allowing direct evaluation of organ function ex vivo, such as bile production and lactate clearance in the liver or urine output in the kidney [90, 91, 92]. Randomized and multicenter studies have shown that NMP increases the utilization of high‐risk donor livers and may reduce the incidence of early allograft dysfunction (EAD), although its long‐term benefits remain under investigation [90, 92]. Hypothermic machine perfusion (HMP)/HOPE uses cold, oxygenated perfusion to limit IRI. HMP reduces the incidence of delayed graft function (DGF) and improves short‐term kidney transplant outcomes [93, 94, 95]. Sub‐normothermic perfusion (sub‐NMP) serves as an intermediate approach, suppressing excessive metabolism while preserving measurable functions for balanced assessment [91, 96]. Organ‐specific adaptations have also developed. EVLP integrates ventilation and perfusion to evaluate gas exchange (PaO_2_/FiO_2_ ratio and compliance) and enables targeted reconditioning, markedly expanding the donor lung pool [97, 98].

#### 5.1.2 Organ‐Specific Applications and Functional Assessment Parameters

Although the principles of EVMP are shared across organs, each graft type requires distinct parameters to assess viability and recovery. Tailoring perfusion conditions and functional markers to specific organs is crucial for precise evaluation and standardization [99, 100, 101] (Table 1).

**TABLE 1:** Viability criteria and key parameters for organ perfusion during machine preservation.

Organ	Perfusion type and modality	Parameters and Physiological Markers	Viability criteria	Purpose	References
Liver	6 h of NMP	Bile production	Cumulative bile production ≥30 g	Assessable biomarker of hepatic viability during EVMP	[102]
Liver	3 h/4 h of NMP	Hepatic artery and portal vein flow	Hepatic arterial flow > 150 mL/min, portal venous flow > 500 mL/min	Reflect the quality of perfusion and the patency of microcirculation in grafts	[103, 104, 105]
Liver	4 h of NMP	Perfusate lactate clearance	Lactate < 2.5 mmol/L	Assesses hepatocellular metabolic function and mitochondrial activity	[104, 106]
Liver	2.5 h of NMP	Biliary bicarbonate, pH, and glucose levels	pH > 7.48, bicarbonate > 18 mmol/L, glucose < 16 mmol/L, and bile/perfusate glucose ratio < 0.67	Accurate biomarkers of bile duct injury (BDI)	[107, 108]
Liver	6 h of NMP	Perfusate AST/ALT release	AST/ALT peak <3000 UI/mL	High levels during perfusion suggest risk of primary nonfunction	[109]
Kidney	4 h of HMP	Perfusate GST isoenzymes (α‐GST and pi‐GST)	α‐GST < 400 U/L and pi‐GST < 60 U/L	Reflect the degree of renal tubular injury	[110]
Kidney	8 h of HMP	Pump parameters (resistance and flow)	Perfusion flow > 40 mL/min and resistance < 0.3 mmHg/mL/min within 1 h	Related to DGF risk and 6‐month estimated GFR (eGFR)	[111]
Kidney	24 h of NMP	Urine output	Urine output > 1 mL/min (remains stable or increases within 6 h)	Reflects the comprehensive functions of glomerular filtration and tubular respiration/secretion	[112]
Kidney	24 h of NMP	Arterial flow, pH, lactate	Arterial flow ≥ 100 mL/min, pH > 7.3, and lactate < 2 mmol/L	Reflection of perfusion kinetics and metabolic status	[112]
Kidney	48 h of NMP	Metabolites and injury markers	NGAL and KIM‐1 did not continuously increase, lactate stable, and urine output maintain	Dynamic monitoring of metabolic homeostasis and injury recovery during long‐term perfusion	[113]
Heart	NMP	Hemodynamic performance	Systolic blood pressure > 60 mmHg, stable heart rhythm, and output flow rate > 1.5–2 L/min	Estimate transplantability through myocardial contractility and overall function (NCT00855712; completed)	[114]
Heart	NMP	Functional parameters	EF < 18%, SW < 1309 mmHg mL, tau > 44 ms	Identify hearts at risk of poor posttransplant function	[115]
Heart	6 h of NMP	OCS Heart arterial lactate and perfusion parameters	Final OCS Heart arterial lactate <5 mmol/L and stable OCS perfusion parameters within recommended ranges	Improve the utilization rate of ECD hearts and reduce the incidence of primary heart graft dysfunction (PGD)	[116]
Lung	4–6 h of EVLP	Oxygenation index	PaO_2_/FiO_2_ > 400 mmHg	Evaluate the transplantability of high‐risk donor lungs after perfusion	[117, 118]
Lung	12 h of EVLP	Lung compliance, airway pressure, and pulmonary vascular resistance	/	Ex vivo PO_2_ may not be the first indication of lung injury, and evaluation of other physiologic parameters takes on greater importance.	[119]
Lung	2–4 h of EVLP	Blood gas analysis of perfusate	ΔPO_2_ > 350 mmHg	Evaluate the improvement of the originally unavailable lungs after EVLP	[120]

In liver preservation, EVMP supports oxygen consumption, promotes lactate clearance, and allows the monitoring of bile and perfusate composition to assess cellular and metabolic integrity. Bile flow, bile pH, and the glucose/lactate ratio serve as early indicators of biliary and hepatocellular recovery, whereas lactate clearance reflects mitochondrial oxidative function. The Organ Care System (OCS) Liver PROTECT trial showed that NMP reduced EAD and nonanastomotic biliary strictures while improving the use of margins, especially in DCD livers (NCT02522871; completed) [92]. HOPE further mitigated reperfusion injury by enhancing mitochondrial respiration and protecting the biliary epithelium [121]. Continuous monitoring of bile and perfusate metabolites has also emerged as a valuable predictor of posttransplant biliary complications [106, 122, 123].

During kidney perfusion, EVMP reduces IRI and promotes faster functional recovery [93]. Functional markers, such as urine output, perfusion resistance, and biomarkers (LDH, GST, NGAL, and KIM‐1) indicate tubular and parenchymal integrity. HMP has been shown to reduce DGF, while extended NMP enables the metabolic repair of marginal kidneys (ISRCTN13292277; completed) [99, 124]. Notably, parameters such as oxygen consumption, lactate clearance, and ATP restoration are strong indicators of mitochondrial energy recovery [125], supporting multiparametric evaluation as a superior method for assessing graft viability [112, 126].

The TransMedics OCS system enables safe preservation beyond 6 h without compromising posttransplant survival [127]. Under NMP, DCD hearts regain spontaneous contractility, significantly expanding the donor pool [128]. Lactate clearance during perfusion is a key indicator of graft transplantability [129]. Although extended perfusion may broaden its clinical applicability, it also carries potential risks of metabolic disturbances and cardiac dysfunction [130]. Moreover, animal studies have demonstrated that plasma exchange and filtration interventions can maintain metabolic stability during 24‐h perfusion [130, 131].

In lung transplantation, EVLP maintains a ventilation–perfusion balance to preserve gas exchange and enable reconditioning. Key parameters include the oxygenation index, lung compliance, and pulmonary vascular resistance [132, 133]. Clinical studies have shown that survival after EVLP‐treated transplantation is comparable to that of standard cold‐stored lungs (NCT01963780; completed) [134]. By modulating perfusate oxygenation levels and monitoring metabolic substrate consumption, EVLP has evolved from a simple assessment tool to an interventional platform for functional optimization [132, 135, 136].

#### 5.1.3 Regenerative and Therapeutic Frontiers of EVMP

EVMP has evolved from a preservation method to a bioengineering and therapeutic platform capable of metabolic reprogramming and organ repair. Its core value lies in achieving metabolic and microenvironmental rebalance through a controllable perfusion setting. Optimizing perfusate composition and oxygenation parameters can alleviate oxidative stress, enhance microcirculation, and promote mitochondrial recovery [137, 138]. These advances demonstrated the feasibility of restoring marginal grafts through metabolic and cellular reconditioning. Moreover, the EVMP platform has emerged as a vehicle for precise interventions and regenerative modulation. Gene vectors, stem cell‐derived exosomes, organoids, and small molecules can be administered locally to achieve organ‐specific modulation without systemic toxicity [139, 140]. For instance, viral vector delivery under NMP resulted in strong, widespread transgene expression in donor hearts, with detectable products lasting up to 5 days posttransplant, while systemic exposure remained minimal [141]. Because perfusion parameters are precisely controllable, EVMP provides a bridge between in vivo and ex vivo experimentation, facilitating mechanistic studies on metabolism, inflammation, and regeneration.

**FIGURE 4:**
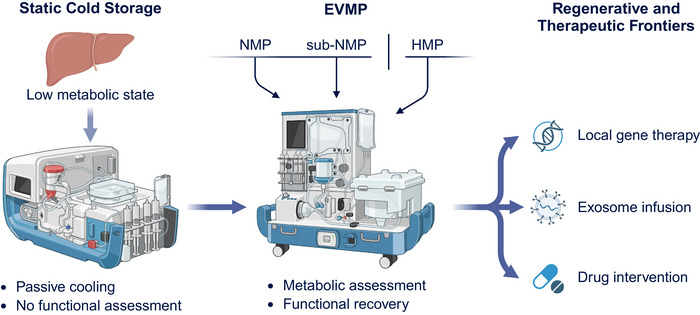
Evolution of ex vivo machine perfusion from preservation to therapeutic bioengineering: integration of metabolic assessment, repair, and regeneration. This schematic illustrates the progressive transformation of organ preservation technology, from static cold storage (SCS) to dynamic, multifunctional ex vivo machine perfusion (EVMP). On the left, SCS represents a low‐metabolic, passive cooling approach without functional assessment. The central panel depicts an EVMP circuit, comprising a pump, oxygenator, reservoir, sensors, and adjustable perfusate flow, operating under three major temperature regimes: hypothermic (HMP), sub‐normothermic (sub‐NMP), and normothermic (NMP). Continuous monitoring of metabolic and hemodynamic parameters enables real‐time organ evaluation and repair. On the right, the diagram highlights the emerging regenerative and therapeutic frontiers, where EVMP serves as a precision platform for localized gene therapy, exosome infusion, and pharmacologic intervention, bridging preservation science with regenerative bioengineering. This figure was created with BioRender (https://biorender.com/).

In summary, EVMP integrates preservation, assessment, and regeneration within a single controllable platform (Figure 4). It enhances the graft quality, reduces IRI, and supports real‐time monitoring. However, major challenges remain, such as the lack of standardized perfusate formulations, hemodynamic parameters, and biomarker thresholds. Future advances should focus on data‐driven multidimensional monitoring integrating metabolomics, transcriptomics, and imaging data using AI‐based predictive systems. The convergence of intelligent control, biomaterials, and regenerative medicine will ultimately transform EVMP into a precise organ medicine platform capable of active repair and remodeling.

### 5.2 Biofabrication in Transplantation: Tissue Engineering and 3D Bioprinting

Biofabrication, the integration of biomaterials, living cells, and advanced manufacturing technologies, such as three‐dimensional (3D) printing, microfluidics, and bioassembly, has emerged as a transformative approach for constructing tissues and organs with defined structures and functions [142, 143]. With rapid advances in materials science, printing precision, and multicellular assembly, biofabrication has transitioned from experimental research to early clinical translation [144, 145, 146]. Transplantation offers an innovative solution to the persistent shortage of donor organs [144, 147], enabling personalized graft design with improved immunocompatibility [148] and reduced rejection risk through the use of autologous cells [149]. Recent breakthroughs in vascularized tissue printing and composite organ fabrication have expanded the potential for clinical application [150]. Recent advances in tissue engineering and 3D bioprinting have improved the prospects for organ transplantation.

#### 5.2.1 Core Technologies and Methodological Advances

Tissue engineering, the foundation of biofabrication, is based on the classical triad of cells, scaffolds, and signaling cues [151]. Stem and progenitor cells, as primary cellular components, possess extensive regenerative capacities and play central roles in tissue repair across multiple organs [152, 153]. Scaffolds, particularly decellularized extracellular matrices and synthetic biomaterials, provide essential structural frameworks [154], while biochemical and mechanical signals guide proliferation, differentiation, and maturation [155]. 3D bioprinting builds on tissue engineering by employing imaging‐derived design data to enable patient‐specific spatially controlled structures of cells, biomaterials, and signaling molecules [156, 157]. It supports predesigned perfusion and replicates the microenvironment of native. These advances have facilitated the fabrication of personalized grafts, recellularization of decellularized scaffolds, and the construction of vascular networks, thereby promoting rapid graft integration and functional recovery [156, 158, 159].

#### 5.2.2 Applications Across Organs

Progress in tissue engineering and 3D bioprinting has been demonstrated in various organ systems [151, 156]. Applications have evolved from patch and conduit reconstruction to functional parenchymal tissue engineering. Recellularized and decellularized scaffolds of the heart and liver have achieved organ‐scale architectures capable of laying the groundwork for anatomically zoned printing‐recellularization strategies [158, 160]. Furthermore, printable and heterogeneous vascularized constructs offer the necessary basis for perfusion and molecular exchange, thereby supporting the survival and maturation of tissue grafts [159]. Clinically, bioengineered airways fabricated from patient imaging data have validated personalized designs and surgical anastomoses, confirming the feasibility of individualized grafts [161, 162]. Collectively, these advances form a closed‐loop imaging–design–biofabrication–validation workflow that accelerates graft integration and functional recovery.

Recent developments are shifting the field from structural mimicry to functional integration, emphasizing multiscale vascularization, innervation, and dynamic microenvironments that deliver bioactive factors in spatially and temporally controlled patterns. Simultaneously, digitalization and AI are increasingly incorporated throughout the process, from imaging to design, printing, and quality control, enabling in situ bioprinting and organ‐on‐chip parallelization for functional testing. Integration with GMP‐compliant materials and standardized protocols further enhances reproducibility and regulatory readiness [151, 155, 156]. Together, these strategies aim to achieve rapid reperfusion, predictable host integration, and personalized reconstruction of patches, tubular structures, and partial organ units, moving biofabrication closer to clinical application.

Despite the remarkable progress, several obstacles hinder the widespread clinical translation of tissue engineering and 3D bioprinting. The long‐term perfusion stability and vascular‐tissue anastomosis of these constructs remain limited. Mechanical mismatches between engineered and native tissues compromise durability; however, the predictability of functional maturation and long‐term remodeling after transplantation remains uncertain. Near‐term clinical translation is most feasible for partial functional units, such as vascular patches, airways, or bile ducts, whereas whole‐organ replacement requires further advances in vascular and neural integration, immune modulation, and large‐scale standardized manufacturing. Overall, the convergence of material innovation, intelligent design, and regenerative biology is expected to drive biofabrication from experimental feasibility to a therapeutic reality, ultimately transforming the future landscape of organ transplantation.

### 5.3 Engineering Immunity: Genetic and Cellular Strategies

**FIGURE 5:**
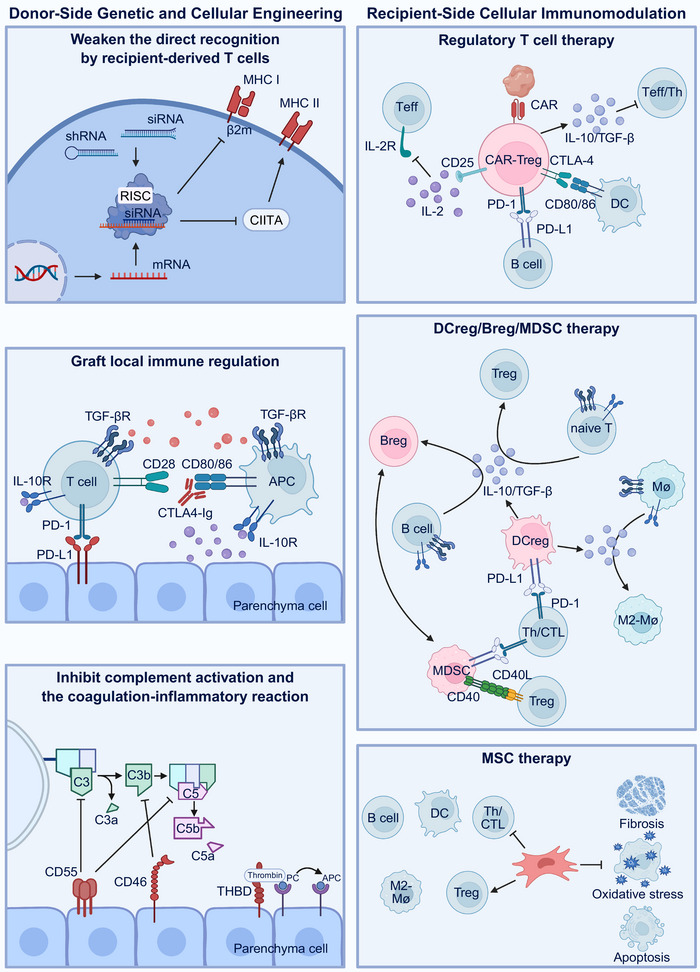
Integrated schematic of donor‐side engineering and recipient‐side cellular immunomodulation strategies for transplant immune tolerance. Left panel: Donor‐side genetic and cellular engineering. (Top) Weaken the direct recognition by recipient‐derived T cells. By using techniques such as siRNA and shRNA to target β2‐microglobulin (β2m) or MHC class II transactivator (CIITA), the expression of MHCI/II was partially reduced, thereby weakening the direct recognition response of receptor T cells. (Middle) Graft local immune regulation. Expression of immunoregulatory molecules, such as PD‐L1, CTLA4–Ig, IL‐10, and TGF‐β, on graft parenchymal or endothelial cells suppresses effector T‐cell activation through PD‐1/PD‐L1 and CD28–CTLA4–Ig signaling axes, promoting local immune quiescence. (Bottom) Inhibition of complement activation and the coagulation–inflammation cascade. Expressing complement‐regulatory proteins (CD46, CD55) or thrombomodulin (THBD) to suppress the complement‐coagulation cascade and mitigate ischemia–reperfusion injury. Right panel: Recipient‐side cellular immunomodulation. (Top) Regulatory T‐cell therapy. Adoptively transferred CAR‐Treg cells stably expressing FOXP3 suppress effector T cells via IL‐10 and TGF‐β secretion, CTLA‐4‐mediated costimulation blockade, and PD‐1/PD‐L1 signaling. Low‐dose IL‐2 support maintains Treg cell lineage stability and therapeutic persistence. (Middle) DCreg/Breg/MDSC therapy. Regulatory dendritic cells (DCreg) expressing PD‐L1 and secreting IL‐10/TGF‐β induce FOXP3+ Treg cells, M2‐like macrophages, and IL‐10+ regulatory B (Breg) cells, while myeloid‐derived suppressor cells (MDSCs) amplify suppression of Th/CTL responses through CD40–CD40L interactions. These populations form an interlinked tolerogenic network reinforcing immune quiescence. (Bottom) MSC therapy. Mesenchymal stem cells (MSC) exert broad immunomodulatory effects by secreting IL‐10, TGF‐β, PGE_2_, and IDO, thereby suppressing Th/CTL and B‐cell activation, inducing Treg cell and M2‐like macrophages, mitigating fibrosis, oxidative stress, and apoptosis, and promoting tissue repair and long‐term graft survival. This figure was created with BioRender (https://biorender.com/).

Engineering Immunity refers to the targeted modulation of the immune system through genetic editing, cellular therapy, and biomaterial‐based approaches to maintain graft function while minimizing systemic immunosuppression [163, 164, 165]. The goal is to induce durable immune tolerance, reduce rejection and infection risks, and prolong graft survival [166, 167]. This strategy acts on both donor and recipient sides, reshaping immunogenicity and immune regulation through precise molecular and cellular interventions (Figure 5).

#### 5.3.1 Donor‐Side Genetic and Cellular Engineering

Recent advances have allowed preconditioning of donor grafts for immune tolerance before transplantation. By silencing key immunogenic genes such as β2‐microglobulin (β2m) or MHC class II transactivator (CIITA) using siRNA or shRNA during ex vivo perfusion, MHC class I/II expression can be reduced, thereby attenuating direct T‐cell recognition. For instance, lentiviral delivery of shβ2m and shCIITA in porcine hearts effectively downregulated SLA I/II expression without inducing inflammation [168]. However, complete MHC loss may activate NK cells that lack self‐responses. To counteract this, selective preservation of nonpolymorphic molecules such as HLA‐E and HLA‐G is often employed [169, 170, 171]. In addition, grafts can be engineered to overexpress immunoregulatory molecules, including PD‐L1, CTLA4–Ig, IL‐10, and TGF‐β, creating a locally tolerogenic environment that promotes T‐cell anergy or exhaustion. In renal transplantation and ex vivo perfusion models, the local expression of membrane‐anchored PD‐L1 reduces inflammatory infiltration and improves early histopathological outcomes [172].

Further strategies include expressing complement‐regulatory proteins (CD46 and CD55) or thrombomodulin (THBD) to suppress the complement‐coagulation cascade and mitigate IRI [173]. Combined with metabolic reprogramming and endothelial stabilization, these interventions reshape the graft microenvironment toward immune quiescence. Importantly, the NMP and HOPE platforms enable these genetic and pharmacological modifications in a controlled, organ‐specific manner, thereby reducing systemic exposure and long‐term uncertainty.

#### 5.3.2 Recipient‐Side Cellular Immunomodulation

Recipient‐side strategies focus on engineering regulatory immune cell lineages to induce graft tolerance, while limiting systemic immunosuppression. Treg cell therapy aims to maintain lineage stability via the FOXP3–TSDR axis and enhance antigen specificity through CAR‐Treg cells [174, 175, 176, 177]. The success of this therapy depends on high‐quality manufacturing and sustained in vivo support with low‐dose IL‐2, which together ensure the efficacy and persistence of Treg cell therapy.

In addition to adaptive immunity, innate immune reprogramming has also gained attention. Tolerogenic dendritic cells (DCregs) generated by conditioning with IL‐10, retinoic acid, or vitamin D3 conditioning promote peripheral tolerance and have demonstrated safety in early clinical trials [81, 178]. Similarly, MSCs exert anti‐inflammatory and tissue repair functions, showing promise in reducing immunosuppressant dependence, although infection and tumor risks remain under investigation [179, 180].

Additional cell types, such as myeloid‐derived suppressor cells (MDSCs) and regulatory B (Breg) cells act as immunoregulatory amplifiers, suppressing effector T‐cell activity and enhancing the tolerogenic milieu, which can synergize with Treg cell or DCreg therapies [181, 182, 183]. Mixed chimerism and thymic tolerance induction further complement these cell‐based strategies by achieving central and peripheral tolerance; however, long‐term stability requires validation in larger trials [184, 185].

#### 5.3.3 Targeted Delivery Platforms for Immune Modulation

Efficient delivery of immunomodulatory agents is crucial for achieving durable tolerance. Recently, EVMP platforms and gut‐targeted delivery systems have emerged as key technologies to achieve this goal. EVMP platforms (NMP and HMP) allow the localized delivery of anti‐inflammatory agents, gene vectors, or cells directly to donor organs [186, 187]. This approach reduces systemic immunosuppression, enables targeted conditioning, and mitigates IRI prior to transplantation. In parallel, gut‐targeted delivery systems, which leverage the gut–immune–microbiota axis, induce peripheral tolerance through mucosal immune mechanisms [188, 189]. These platforms achieve systemic donor‐specific tolerance with minimal toxicity by directing the antigen exposure to the intestinal immune system. The two strategies are complementary. EVMP platforms focus on pretransplant graft conditioning through local microenvironmental modulation, whereas gut‐targeted systems emphasize systemic or mucosal tolerance induction. Together, these systems reduce dependence on long‐term systemic immunosuppressive therapy and provide a dual safeguard for advancing clinical tolerance strategies.

### 5.4 AI‐Driven Transplantation: Predictive Analytics and Precision Medicine

AI is rapidly evolving from a supplementary tool to a central hub for decision support in transplantation medicine. By integrating complex, multisource medical data, AI provides valuable insights into donor–recipient matching, organ allocation, postoperative risk prediction, and personalized immunosuppressive management [190, 191, 192, 193]. The ability of AI to learn from large datasets and uncover latent patterns enhances organ utilization, reduces the risk of graft failure, and supports precision immunosuppressive therapy, ultimately improving patient survival and transplant outcomes. Methodologically, transplantation‐focused AI applications require the tailored use of temporal models, imaging analytics, and multiomics integration frameworks to collectively support clinical decision‐making [194, 195, 196, 197].

#### 5.4.1 AI in Donor–Recipient Matching and Organ Allocation

AI‐based platforms have shown considerable promise in enhancing donor–recipient matching and improving organ allocation processes. For instance, in kidney transplantation, the platform Nephron, based on a gradient boosting (GB) classifier, achieved a 99% accuracy rate in multicenter retrospective datasets, far surpassing traditional rule‐based matching systems [197]. This system captures complex, nonlinear relationships between donor and recipient features and offers scalable solutions for clinical organ allocation. However, despite its high accuracy, concerns regarding overfitting of training datasets persist. Further, prospective multicenter studies are necessary to validate the generalizability and performance of these platforms in diverse patient populations [197].

In contrast, Guijo‐Rubio et al. compared machine learning models with traditional logistic regression for predicting donor–recipient matching [198]. Surprisingly, logistic regression outperformed machine learning models in predicting 5‐year survival outcomes. This highlights the fact that the effectiveness of AI is highly dependent on factors such as data quality, sample size, and variable selection, underscoring the need for robust model validation.

#### 5.4.2 AI in Postoperative Risk Prediction and Immunosuppressive Management

In postoperative care, AI has proven invaluable for predicting the risk of graft failure and optimizing immunosuppressive therapy. For example, the iBox prognostic system integrates renal function metrics, immunological features, and biopsy pathology to assess the risk of graft failure across multiple posttransplant time points [199]. This system has been validated in international multicenter cohorts, and is now a standard tool for risk stratification and follow‐up management.

Furthermore, AI‐based models such as those developed by Yoon et al. for tacrolimus blood concentration prediction have shown great promise for personalized immunosuppressive management [196]. By forecasting the next‐day drug levels using postoperative monitoring data, these models improve drug dose precision and reduce the risks of toxicity and overimmunosuppression. Similarly, XGBoost algorithms have been used to predict tacrolimus concentrations in kidney transplant recipients, demonstrating better accuracy than traditional Bayesian models [200]. These models effectively shortened the dosage adjustment cycle and reduced the risk of drug toxicity. However, their performance depends heavily on the quality of the input variables, and may exhibit amplified prediction errors when confronted with incomplete or noisy data [201].

#### 5.4.3 AI in Organ Evaluation and Multiomics Integration

The ability of AI to process and analyze high‐dimensional data has significant applications in organ evaluation. For example, the InsighTx model, developed to analyze EVLP data, uses metabolic, ventilatory, and hemodynamic parameters to predict posttransplant organ function [191]. This AI model helps determine transplant suitability early, improves organ utilization, and shortens the evaluation time.

Moreover, the integration of multiomics data, including genomic, transcriptomic, and proteomic information, with clinical and perfusion data has enhanced the prediction of rejection events and transplant outcomes. A systematic review of 29 machine learning models for kidney transplantation revealed the ability of AI to capture complex nonlinear relationships among various factors, thereby significantly enhancing graft survival predictions [202]. This conclusion is further supported by emerging integrative multiomics studies that combine clinical, transcriptomic, proteomic, and single‐cell features to markedly enhance the prediction of rejection events and transplant outcomes [203].

In addition, our group constructed the first single‐cell immune atlas for liver transplantation, which systematically reveals the heterogeneity and functional lineages of immune cells at single‐cell resolution [204]. Notably, a subset of CD8+ tissue‐resident memory T cells has been identified as critical mediators of rejection [205]. Collectively, these studies provide a high‐resolution immunological foundation for AI models that integrate multiomics and single‐cell data, thereby facilitating the development of interpretable predictive models for transplant immunology.

Despite significant advancements, several challenges remain regarding the integration of AI into clinical transplantation. One major obstacle is model interpretability because AI systems are often viewed as black boxes, making it difficult to understand how they arrive at predictions. This lack of transparency can hinder widespread adoption in clinical practice. Furthermore, data standardization remains a critical issue. AI models in transplantation rely on high‐quality, standardized datasets; however, the heterogeneity of data sources, including clinical records, imaging, and multiomics, complicates model training and validation. Looking ahead, multicenter validation studies and ethically governed AI systems are crucial for refining AI‐driven transplantation tools. The goal is to achieve seamless integration of predictive models, real‐time data streams, and clinical workflows, enabling a more personalized, data‐driven approach to organ transplantation.

## 6 Xenogeneic Organ Transplantation

Xenogeneic transplantation has progressed from a speculative concept to a rapidly advancing frontier, driven by breakthroughs in source‐animal engineering, immunomodulatory strategies, and multigene‐edited donor lineages. These developments aim to bridge the widening gap between organ supply and demand by creating physiologically compatible, virologically safe, and immunologically optimized porcine grafts. Parallel efforts have focused on mitigating infectious and zoonotic risks and establishing robust regulatory and ethical frameworks to ensure responsible clinical translation. Together, these advances outline a comprehensive roadmap toward making xenotransplantation a viable complement to human organ donation while highlighting the scientific, immunological, and societal hurdles that must be addressed before routine clinical application becomes possible.

### 6.1 Source‐Animal Engineering

The persistent shortage of organ donors has positioned source‐animal engineering as a cornerstone for advancing xenotransplantation toward clinical use [206]. This strategy employs gene‐editing technologies to delete porcine genes encoding carbohydrate antigens that trigger major human immune responses, such as GGTA1, CMAH, and B4GALNT2, while introducing human immunoregulatory, anticoagulant, and anti‐inflammatory proteins, including CD46, CD55, and THBD. These modifications enhance endothelial protection and reduce complement and coagulation. Additionally, inactivation of porcine endogenous retroviruses (PERVs) further reduces the risk of cross‐species viral transmission [207]. The integration of highly efficient gene‐editing platforms such as CRISPR/Cas9 with somatic cell nuclear transfer (SCNT) has enabled the generation of multigene‐edited pigs, markedly improving xenograft safety and functional compatibility [208, 209, 210]. Although nonhuman primates share a high immunological similarity with humans, their use is restricted by ethical and zoonotic concerns. In contrast, pigs offer anatomical and physiological compatibility, are amenable to large‐scale breeding, and have well‐established gene editing platforms [211, 212]. Consequently, research has focused on multigene engineering strategies that optimize porcine immunological and physiological traits to create clinically viable donor lineages.

#### 6.1.1 Gene/Phenotype Target Design Strategy

Target design is central to source‐animal engineering and requires a coordinated approach that reduces immunogenicity, aligns complement and coagulation compatibility, and preserves organ structure and function [206]. Recent studies have demonstrated that multigene high‐precision editing combined with rigorous phenotypic validation is essential for success in preclinical xenotransplantation models [173, 213].

The first target category involves the deletion of carbohydrate antigens responsible for AMR, including GGTA1, CMAH, B4GALNT2 [208, 214]. These multigene knockouts substantially decrease human and nonhuman primate antibody binding to xenografted tissues, thereby limiting complement activation and immune injury [215]. In brain‐dead, human models, kidneys from pigs with a triple gene knockout and transgenic expression of hCD55 and hTBM maintained favorable graft function, although acute rejection still occurred in some cases [216].

The second category includes human complement‐regulatory, anticoagulant, and antithrombotic proteins. Transgenic expression of CD46, CD55, and CD59 suppresses excessive complement activation [217, 218], whereas the expression of hTBM, endothelial protein C receptor, tissue‐factor‐pathway inhibitor, and CD39 prevents thrombotic microangiopathy and endothelial injury [219, 220]. Notably, multitransgenic pig organs have shown extended survival and improved function in nonhuman primate models [221, 222, 223].

The third category focuses on the immune modulation. Expression of human CD47 interacts with signal‐regulatory protein α (SIRPα) on macrophages to suppress phagocytic signals, protecting grafts from innate immune attack. In pig‐to‐nonhuman primate kidney transplantation, glomerulus‐specific hCD47 expression reduced proteinuria [224]. Similarly, in porcine red blood cell (pRBC) modification studies, triple‐knockout pRBCs coexpressing hCD47 and hCD55 exhibited prolonged circulatory persistence and reduced antibody binding following transfusion into rhesus macaques [225].

Additional targets include the anti‐inflammatory, antioxidative, and antiapoptotic pathways [226]. Genes, such as human heme oxygenase‐1 and A20, enhance xeno‐graft tolerance to IRI and chronic inflammation [227, 228]. Moreover, combining these with complement‐ and coagulation‐modifying genes produces synergistic provides, resulting in improved xenograft survival and function [229, 230].

#### 6.1.2 Advances in Multiplex Genome Engineering Platforms for Source‐Animal Optimization

Multiplex gene editing and transgenic integration have become dominant engineering strategies for overcoming the complex immunological, coagulative, and virological barriers of xenotransplantation. Early single‐gene knockouts are insufficient for durable graft survival. Thus, researchers have now pursued combinatorial approaches that achieve both multigene deletion and human gene insertion [209, 231]. The CRISPR/Cas system offers high efficiency for multiplex editing. Coselection markers, such as antibiotic‐resistance genes or fluorescent reporters, to enrich for high‐quality edited clones, which are then used in SCNT or embryo microinjection to produce genetically modified pigs [232, 233]. Recently, porcine expanded potential stem cells (pEPSCs) have emerged as a flexible and stable platform that simplifies workflow and increases editing efficiency.

Donor pig genome engineering has evolved from sequential gene stacking to simultaneous, high‐throughput, and multilocus precision editing. Multi‐guide RNA designs, high‐fidelity Cas variants, and near‐PAM‐independent nucleases have expanded the editable regions while minimizing off‐target effects [234, 235, 236]. Coselection and surrogate reporter systems further enhance the editing efficiency and homozygosity, enabling the generation of clones with up to seven loci edited in one round [237]. Genome‐wide PERV inactivation has been achieved both in vitro and in vivo [238], and next‐generation precision editors, such as base and prime editors, allow allele humanization without DNA double‐strand breaks, thereby improving safety and controllability [239, 240].

Technological innovations have shifted donor‐animal engineering from empirical gene stacking to systematic and predictive frameworks. The modular design–build–test–learn cycle now guides the assembly of regulatory modules and evaluation through multiomics and single‐cell analyses. Long‐read sequencing technologies (PacBio HiFi; Oxford Nanopore) and whole‐genome validation pipelines ensure genome integrity and accurate transgene insertion [241]. Notably, pEPSCs provide a robust genomic chassis for reproducible high‐quality donor design [242]. Collectively, these advances have transformed donor animal engineering into a modular, standardized, and scalable system that underpins the clinical translation of xenotransplantation [173, 210].

### 6.2 Preclinical and Early Clinical Experience

Preclinical xenotransplantation research relies primarily on two models: the pig‐to‐nonhuman primate (NHP) model and the pig‐to‐human cadaver (brain‐dead) recipient model. The former is used to evaluate immune rejection and physiological integration, whereas the latter focuses on clinical safety factors such as cross‐matching, perfusion dynamics, and early complications under human physiological conditions [212, 243, 244]. Pigs have become the preferred donor species because of their anatomical compatibility, short reproductive cycles, and mature gene‐editing systems. These include xenogeneic antigen knockouts (GGTA1, CMAH), expression of human complement and coagulation regulators (hCD46, hTBM), and inactivation of PERVs. In NHP models, multigene‐edited porcine organs have achieved significantly extended survival, with orthotopic heart transplants surpassing 6 months and 10‐gene‐edited kidneys maintaining long‐term function under clinical immunosuppression protocols [212, 244, 245, 246]. These results provide essential data for the design of future clinical trials.

Preclinical studies typically combine in vitro and in vivo validations. In vitro experiments, including serological cross‐matching, complement and coagulation interaction tests, and single‐cell or spatial genomic analyses allow for the rapid assessment of antigenicity and immune activation [245]. In vivo validation using orthotopic or life‐support transplantation models in NHPs evaluates physiological integration, such as filtration, endocrine, and metabolic functions, as well as rejection patterns [212, 244]. Brain‐dead human models complement these studies by assessing surgical feasibility, perfusion behavior, and perioperative complications, thereby providing a safety reference for eventual clinical trials [243]. Although rodent and humanized‐mouse models remain valuable for mechanistic studies, their findings require confirmation in large animal or cadaver systems for translational relevance [246].

However, immune rejection remains a major barrier to xenotransplantation. Acute rejection commonly results in rapid graft failure, driven by preformed antibodies against xenogeneic carbohydrate antigens [245]. Genetic modification of donor pigs, particularly GGTA1 and CMAH knockouts, has effectively reduced antibody recognition and complement activation [210, 238], significantly improving the compatibility between porcine organs and the human immune system [245]. In addition to genetic engineering, progress in immunosuppressive protocols is pivotal. The combination of anti‐CD40 antibodies, costimulatory‐signal inhibitors, and IL‐2R antagonists effectively regulates T‐cell activation and promotes long‐term graft survival in NHP models [243, 244]. Additionally, complement inhibitors have been incorporated to address thrombotic microangiopathy, an issue unique to xenotransplantation. These optimized immunosuppressive protocols have significantly reduced the incidence of acute rejection, thereby providing a critical pharmacological foundation for the clinical translation of xenotransplantation.

The two major indicators of xenotransplantation success are graft survival time and physiological function. Advances in multigene‐edited donor pigs and optimized immunosuppressive regimens have markedly extended graft longevity in NHPs. In kidney xenotransplantation, survival times exceeding 300 days have been achieved while maintaining near‐normal glomerular filtration, electrolyte balance, and endocrine function [212, 244]. Similarly, orthotopic heart transplants from genetically modified pigs sustained cardiac activity beyond 6 months, maintaining stable hemodynamics and electrocardiographic rhythms [245, 247]. Research on other solid organs, such as the liver, is also progressing. In brain‐dead human models, gene‐edited pig livers demonstrate short‐term human albumin synthesis and bile secretion, confirming metabolic activity and viability [248, 249].

Building upon preclinical achievements, xenotransplantation has entered early clinical exploration. Recent pilot trials have used multigene‐modified pigs with antigen knockouts and human protective gene insertions [245, 250]. In 2022, the University of Maryland Medical Center performed the world's first live human xenotransplant, transplanting a gene‐edited pig heart into a patient who survived for approximately 2 months postsurgery. Postmortem analysis suggests that graft failure is associated with porcine cytomegalovirus reactivation and immune‐mediated injury [250, 251]. Despite these outcomes, this study marked a milestone in bridging animal studies and human applications. In xenogeneic kidney transplantation, brain‐dead human trials at the University of Alabama at Birmingham and New York University Langone Health demonstrated that multigene‐edited pig kidneys‐maintained urine output, filtration function, and electrolyte regulation without HAR or severe infection [243, 252, 253]. These findings provide crucial safety data for future trials involving living recipients.

Preclinical and early clinical studies have significantly advanced xenotransplantation, improving our understanding of immune rejection, graft physiology, and safety. Multigene editing enhances immune compatibility, whereas optimized immunosuppression extends graft survival. However, several key challenges remain including long‐term immune tolerance, thrombotic complications, and potential viral infections. Ethical considerations, cross‐species immune predictability, and long‐term organ functionality remain the major hurdles in clinical translation.

### 6.3 Immunological Hurdles Unique to Xenografts

Despite remarkable progress in gene‐editing technologies that reduce xenogeneic antigen expression, xenotransplantation continues to face formidable immunological barriers [210, 254]. Even with multigene modifications, recipients mount robust immune responses involving both innate and adaptive mechanisms. Compared with allotransplantation, xenotransplantation elicits a more complex immune cascade encompassing natural antibodies, complement activation, cellular immunity, and inflammation [255]. Understanding these multilayered interactions is essential for designing targeted immunomodulatory strategies. Existing evidence indicates that the immune barriers in xenotransplantation primarily involve host pre‐existing natural antibodies recognizing xenogeneic antigens, abnormal activation of the complement cascade, cross‐species responses of innate immune cells, and the subsequent adaptive immune attack triggered by these interactions [256, 257].

#### 6.3.1 Xenograft Rejection Types

Xenograft rejection can be categorized into HAR, delayed xenograft rejection (DXR), and chronic xenograft rejection (CXR) according to its onset, mechanism, and pathology [254, 256]. HAR typically occurs within minutes to hours after transplantation, triggered by pre‐existing natural antibodies that recognize xenogeneic antigens and activate the classical complement pathway. This can result in endothelial damage and microvascular thrombosis [258]. If partially controlled, the process may progress to DXR, which is characterized by persistent antibody deposition, residual complement activation, and infiltration of monocytes, NK cells, and T cells, along with the release of numerous inflammatory cytokines [255, 258, 259]. In long‐term survivors, chronic inflammation and vascular remodeling may lead to CXR manifesting as intimal hyperplasia and interstitial fibrosis [258].

**FIGURE 6:**
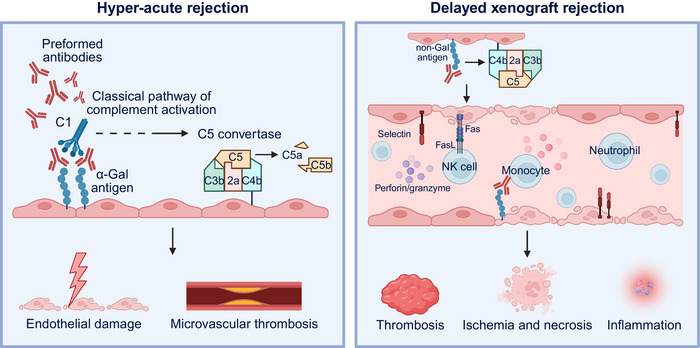
Mechanisms of hyperacute and delayed xenograft rejection. Left panel: Hyperacute rejection (HAR). Preformed natural antibodies in the recipient recognize xenogeneic carbohydrate antigens, most prominently α‐Gal, on donor endothelial cells, activating the classical complement cascade. Complement components C1 and C5 convertase drive C5a/C5b generation, leading to endothelial injury, platelet aggregation, and microvascular thrombosis. These events cause rapid graft failure within minutes to hours after transplantation. Right panel: Delayed xenograft rejection (DXR). Following partial control of HAR, antibody deposition against non‐Gal antigens sustains complement activation and triggers endothelial activation. Upregulated adhesion molecules facilitate monocyte and NK‐cell infiltration. NK‐cell‐mediated cytotoxicity and cytokine release promote thrombosis, ischemia, necrosis, and inflammation within graft vasculature. DXR typically manifests within days to weeks and represents a continuum from early antibody‐ and complement‐mediated injury to mixed cellular participation. This figure was created with BioRender (https://biorender.com/).

Multigene editing in donor pigs has substantially reduced HAR by eliminating major carbohydrate antigens, such as α‐Gal. However, residual low‐titer antibodies, incomplete complement regulation, and cross‐species coagulation incompatibility can cause endothelial activation and localized vascular injury. Current strategies focus on early humoral control by combining antibody depletion methods (plasma exchange or antigen‐specific adsorption) with complement inhibition using C5 inhibitors, soluble complement receptors, or human complement regulatory proteins expressed in the donors. These interventions aim to limit immune coagulation interactions during early reperfusion [260, 261, 262, 263]. Once HAR is mitigated, the rejection often transitions to DXR within a few days or weeks. DXR involves both humoral and cellular immune responses, where induced antibodies against non‐α‐Gal antigens (such as Neu5Gc, SDa) form immune complexes in graft vasculature, sustaining complement activation. Activated endothelial cells express adhesion molecules, such as E‐selectin, ICAM‐1, and VCAM‐1, promoting leukocyte infiltration and cytokine release that amplify vascular inflammation. Interventions at this stage include cellular immune suppression and inflammation blockade, such as costimulatory pathway inhibition, immune checkpoint modulation, and suppression of NK/macrophage activity [264, 265, 266]. HAR and DXR are two stages of the continuous immune spectrum (Figure 6). Early endothelial injury creates a proinflammatory microenvironment that drives DXR progression [267, 268]. Thus, dual strategies combining gene editing with immunomodulation, including tolerance induction, thymus transplantation, or mixed chimerism, are being explored to achieve long‐term graft acceptance [254, 269].

CXR reflects sustained low‐grade immune activation and tissue remodeling imbalance. It features endothelial‐to‐mesenchymal transition (EndMT), smooth muscle migration, and fibrosis driven by chronic inflammation and metabolic stress. Emerging evidence suggests that donor cell senescence and residual antigen exposure further sustain vascular remodeling and functional decline, underscoring the need for long‐term immune regulation strategies [257, 270, 271].

#### 6.3.2 Xenospecific Immune‐Coagulation Coupling and Chronicity

The core immunopathology of xenograft rejection arises from the coupling of immune and coagulation systems, driven by residual xenogeneic antigens [272, 273]. Following deletion of α‐Gal, tissue injury is mainly caused by non‐Gal antigens that elicit antibody and complement activation. Binding of C1q to antigen–antibody complexes triggers complement‐dependent cytotoxicity and generates C3a and C5a fragments that recruit leukocytes and amplify inflammation. When compounded with IRI, these processes enable the progression from HAR to DXR, despite the early control of acute rejection [255, 257]. At the innate immunity level, incompatibility in the CD47–SIRPα axis enhances macrophage phagocytosis and proinflammatory activity, while the absence of inhibitory ligands, such as human HLA‐E or HLA‐G on porcine endothelial cells, lowers the activation threshold for NK cell cytotoxicity [274]. Neutrophil extracellular traps and immediate blood‐mediated inflammatory response, which are commonly observed in islets or cell xenotransplantation, further intensify vascular inflammation and thrombosis [268]. The adaptive immune response is primarily mediated by human T cells, which indirectly recognize porcine antigens. Tfh cells promote antibody affinity maturation and isotype switching against non‐α‐Gal targets, while microbe‐sensitized memory T cells exhibit cross‐reactivity, lowering the activation threshold for secondary immune responses [259].

Organ‐specific immune features also influence xenograft outcomes. The lungs exhibit high sensitivity to complement‐neutrophil interactions, often leading to early permeability edema and microthrombosis [275, 276]. The liver tends toward platelet clearance, coagulation dysfunction, and partial immune tolerance [277], whereas the heart and kidney typically display endothelial inflammation with C4d deposition and microvascular injury [257, 278, 279]. Additionally, persistent antigen exposure sustains chronic inflammation and promotes epitope spreading, plasma cell longevity, and progressive vascular remodeling. Mechanisms such as EndMT, metabolic stress, and cellular senescence collectively lead to xenograft‐specific chronic vasculopathy characterized by intimal thickening and fibrosis [1, 280, 281].

In summary, xenotransplantation rejection involves a dynamic network of natural antibodies, complement activation, and innate, and adaptive immune responses. Although gene‐editing markedly reduced the antigenic load and controlled HAR, immune activity persisted during the DXR and CXR stages. Current immunomodulatory strategies focus on suppressing humoral and cellular responses, and regulating immune checkpoints to delay injury. Future research should elucidate the interplay between these immune pathways and develop integrated interventions to achieve durable xenograft tolerance.

### 6.4 Infectious Risk, Zoonosis Control, Regulatory, and Ethical Considerations

As xenotransplantation advances in clinical applications, the risk of infection remains a critical challenge. These risks arise in both donors and recipients. Donor‐derived infections include exogenous pathogens that can be screened, and endogenous pathogens that are integrated into the genome. PERVs are of particular interest because of their potential to infect human cells. The risk of viral activation is exacerbated by reperfusion injury and immune dysregulation during transplantation [282, 283]. In recipients, long‐term immunosuppressive therapy, which is essential for preventing rejection, increases susceptibility to opportunistic infections and can obscure typical clinical presentations, complicating diagnosis and management [284, 285]. To address these issues, international guidelines recommend a multilayered infection prevention framework for early clinical trials. These include maintaining specific pathogen‐free (SPF) donor herds to prevent exogenous transmission, applying gene‐editing technologies to inactivate PERVs, and implementing continuous posttransplant pathogen surveillance [285, 286].

#### 6.4.1 Zoonotic Risk Management and Biosecurity Measures

Biosafety management is the cornerstone of the sustainable and ethical development of xenotransplantation [287, 288]. Current international standards emphasize the need for stringent donor control and continuous monitoring of pathogens to minimize cross‐species transmission. Stewart et al. proposed that all clinical xenotransplantation should employ closed, SPF‐certified donor pig populations with comprehensive longitudinal pathogen tracking [285]. Similarly, Fishman et al. emphasized synchronized surveillance systems between donor facilities and clinical centers to ensure the traceability and early detection of viral transmission [283]. Public Health Service guidelines also designate donor‐source control and ongoing monitoring as fundamental safety pillars. Establishing and maintaining SPF herds require long‐term closed breeding, multilevel isolation, routine pathogen screening, and genetic lineage verification. Any suspicious virological signal must trigger immediate quarantine and revalidation [282].

At the molecular level, advanced gene‐editing tools enable multilocus PERV inactivation, effectively minimizing the likelihood of cross‐species viral transmission [238, 289]. Cells derived from PERV‐inactivated donors show no infectivity in human cell lines, thus providing a feasible route for biosafe donor populations. However, genomic inactivation by itself cannot replace conventional biosecurity monitoring. It must be integrated with multipathogen detection systems capable of identifying both known and novel agents [290, 291]. To ensure closed‐loop biosafety management, a traceable surveillance network linking donor facilities and clinical centers is required. The system should support data sharing, dynamic risk evaluation, and pathogen traceability. Both the US FDA and the WHO recommend long‐term posttransplant monitoring, biobanking of donor and recipient samples, and transparent reporting mechanisms. Therefore, infectious‐risk control must proceed in parallel with ethical oversight to safeguard public health and foster societal acceptance of xenotransplantation [292, 293].

#### 6.4.2 Regulatory and Ethical Considerations

The clinical translation of xenotransplantation depends not only on scientific advances but also on robust regulatory and ethical frameworks. International regulatory bodies classify xenotransplantation as a high‐risk biological intervention subject to standards comparable to those governing cell and gene therapies. Comprehensive oversight should include full donor traceability, standardized pathogen screening archives, long‐term recipient follow‐up, and biorepository systems for sample storage and future reference [292, 294]. Guidelines further require real‐time data tracking, emergency termination mechanisms, and prompt reporting of any public health events, such as zoonotic outbreaks [295].

From an ethical perspective, ongoing debates focus on the justification for animal use, recipient risk‐benefit balance, and transparency of research processes. The use of animal‐derived organs must adhere to the principles of the 3Rs (replacement, reduction, and refinement) and be limited to research with strong scientific and clinical justification [296]. For recipients, the informed consent process must fully disclose long‐term infection risks, the scope of data sharing, and potential societal impacts. Furthermore, institutional ethics committees should oversee each study phase and conduct continuous ethical evaluations and public communication to maintain accountability and trust [297, 298].

## 7 Ethical, Societal, and Regulatory Issues

Since its first successful implementation in the mid‐20th century, organ transplantation has evolved from a pioneering surgical technique to a complex healthcare system deeply integrated with AI, gene editing, regenerative medicine, and transnational collaboration. It not only represents a major breakthrough in modern medicine, but also reflects the evolving trajectory of social ethics, legal systems, and public trust. Since Professor Murray completed the first kidney transplantation between identical twins in 1954 [299], every transformation in transplant medicine has been accompanied by profound debates over core ethical issues, including standards for determining death, the use of immunosuppressants, and the fairness of organ allocation [300, 301]. With the continuous expansion of donor sources and indications for transplantation, the original ethical framework faces ongoing pressure to reconstruction [302].

Moreover, the scarcity of organ resources and significant value that transplantation provides has rendered related ethical issues urgent and far‐reaching. From the mortality risk of patients on waiting lists, informed consent of donors, risks for living donors, to the challenges of cross‐border transplantation and organ commercialization, the ethical focus of this field has long transcended the traditional relationship between an individual recipient and donor, shifting instead toward systemic issues at the level of society, institutions, and resource distribution [303]. The deep involvement of sociocultural contexts, religious beliefs, and global economic inequalities demands that organ donation systems not only meet medical standards, but also align with public value expectations and demonstrate institutional legitimacy [304, 305].

In the 21st century, technological breakthroughs have rendered the ethical landscape of organ transplantation increasingly complex. Advances in composite tissue allotransplantation, facial transplantation, xenotransplantation, and genetically edited organs have transformed medical advances into complex social issues. These developments prompted a reassessment of whether technological progress, while enhancing clinical outcomes, might erode fairness in the allocation of medical resources. Moreover, data‐driven algorithms raise concerns about their potential to reinforce or even exacerbate existing social biases. Within the donor–recipient relationship, questions persist regarding whether the structure of rights is truly symmetrical [306]. Finally, from the perspective of regulation and governance, the traditional nation‐centered legal framework is gradually evolving toward a model of globalized governance and interdisciplinary collaboration. The development of organ sharing networks, transnational regulatory coordination, and mechanisms for building public trust have become unavoidable topics in contemporary debate. However, in the ongoing race between institutional reform and technological innovation, the institutional lag, ethical ambiguity, and public skepticism constitute three risks that cannot be ignored.

### 7.1 Core Ethical Dilemmas and Societal Concerns

#### 7.1.1 Resource Allocation and Informed Consent: The Dual Challenge of Scarcity and Autonomy

Organ scarcity has long been a fundamental ethical dilemma in the field of transplant medicine, centered on how to strike an appropriate balance between maximizing utility and ensuring fairness in distribution. Persad et al. proposed a classic ethical framework that categorizes allocation principles into four types: equal treatment, priority to the worst‐off, maximization of total benefit, and promotion of social usefulness [307]. This model emphasizes the necessity of a mixed principal approach to prevent the systemic injustice that may arise from adherence to a single standard. In clinical practice, models, such as the MELD score used in liver transplantation and the EPTS model applied in kidney transplantation, seek to enhance the efficiency of organ allocation through objectified and quantitative algorithms. However, excessive reliance on such models may introduce implicit biases. For instance, patients with a higher socioeconomic status often have better access to comprehensive medical evaluations and consistent postoperative follow‐ups, thereby presenting a potential advantage in allocation [308, 309]. Consequently, promoting transparency in the weighting of allocation systems, conducting regular audits of predictive models, and involving public representatives in oversight have become key directions for the ethical governance of modern organ transplantation.

The ethical orientations of different societies become clearly distinguishable in their approaches to opt‐in and opt‐out consent for organ donation. The presumed consent (opt‐out) system adopted in several European countries, has been credited with improving donation rates. However, empirical evidence suggests that its effectiveness depends more on the maturity of the healthcare system and level of public trust than on the legal reform itself [310]. A mixed‐methods study further indicated that, without well‐trained coordinators and sustained public communication, the positive impact of an opt‐out policy on donation rates is often transient [311]. However, the role of family members in donation decisions remains debated. In most countries, when the deceased's prior wishes are unknown, families are granted veto rights. Proponents view this as a gesture of respect for familial grief and relational ethics, whereas opponents argue that it undermines the legal validity of individual autonomy [312]. This tension illustrates the complex and ongoing interplay between personal autonomy and communal values in the profound moral domain of organ donation, the ultimate gift of life.

#### 7.1.2 Equity, Commercialization, and Global Justice: From Individual Ethics to Structural Ethics

Equity in transplantation has evolved from a clinical concern to a central issue of social justice. Studies have consistently demonstrated that socioeconomic status and racial background significantly influence access to transplant waiting lists and the likelihood of receiving transplants. For example, a multicenter study in the United States confirmed that African‐American patients have a markedly lower rate of living donor kidney transplantation than other racial groups, even after adjusting for clinical indications [313]. Similar inequities are more pronounced in low‐ and middle‐income countries, where infrastructure limitations, inadequate health insurance systems, and sociocultural barriers are pervasive. Therefore, the scope of ethical governance must extend from individual fairness to structural justice. This can be achieved by developing standardized equity assessment indicators, harmonizing clinical evaluation procedures, and promoting cross‐institutional data interoperability to mitigate systemic bias.

Organ commercialization and transplant tourism remain among the most destructive issues in global transplant ethics. The Istanbul Declaration of 2008 explicitly condemned all forms of organ trade and related transplant tourism, emphasizing that such practices violated medical ethics and fundamental human rights, and called on nations to establish self‐sufficient donation systems and strengthen international collaboration [314]. In 2010, the WHO further reinforced these principles in its Guiding Principles on Human Cell, Tissue, and Organ Transplantation, highlighting the central importance of voluntary and unpaid donations, transparency, and traceability [315]. However, in regions characterized by economic disparities and weak regulatory oversight, organ trafficking continues to occur in concealed forms. Future governance should shift from purely prohibitive approaches to comprehensive prevention and remediation strategies. This includes promoting cross‐border data sharing, establishing third‐party reporting mechanisms, and providing support and compensation to exploited donors.

From the perspective of global justice, organ shortage is not only a medical challenge, but also a manifestation of global developmental inequality. Ethically, high‐income countries bear the responsibility of assisting resource‐limited regions in developing localized donation systems and organ preservation capacities, rather than alleviating their own shortages through cross‐border procurement. Regional organ sharing networks, such as Eurotransplant, have demonstrated a feasible model grounded in reciprocity and procedural transparency, providing an important reference for equitable international collaboration [316].

#### 7.1.3 Technological Frontiers and Algorithmic Ethics: Explainable Fairness in the Age of AI

AI and predictive analytics are gradually transforming the landscape of organ allocation, matching, and postoperative risk assessment. However, insufficient algorithmic transparency and potential biases embedded within the training datasets have reignited the tension between efficiency and fairness in this new technological context. Although the public generally supports the involvement of AI in liver allocation decisions, such acceptance is conditional on algorithmic interpretability, physician oversight throughout the decision‐making process, and the preservation of an effective mechanism for human review [317].

The core ethical challenge of algorithmic governance lies in the fact that bias often stems from structural inequalities embedded in historical data, rather than intentional design. For instance, if a predictive model is primarily trained on data derived from a single, privileged population, it may systematically underestimate the potential benefits of transplantation in other demographic groups. Currently, ethical oversight of AI in medical applications must shift from abstract principles to operational mechanisms. These include establishing systems for data representativeness review and bias auditing, defining concrete standards for model interpretability, providing patients with clear channels for appeal and human intervention, strengthening public participation, and cross‐institutional regulatory coordination [318]. In the context of transplantation, this necessitates the establishment of a well‐defined ethical framework for human–AI collaboration. AI should serve as a quantitative decision support‐tool, while the ultimate clinical authority and responsibility must remain within human medical teams. Furthermore, international regulations on data governance and privacy protection must evolve in parallel with medical ethics to ensure that the advancement of AI in transplantation promotes, rather than undermines, distributive justice.

### 7.2 Policy Tools and Implementation Mechanisms

The effective implementation of ethical principles depends on their institutionalization into actionable policy instruments. Only when ethical norms are deeply integrated with regulatory systems, data governance, and public trust mechanisms can organ allocation frameworks maintain a sustainable balance between technological innovation and societal legitimacy. Ethical review and compliance oversight form the cornerstone of standardized governance. In both research and clinical trials, research ethics committees and independent data safety monitoring boards play central roles in the approval, risk assessment, and continuous supervision. In high‐risk areas such as living donations and xenotransplantation, international regulatory practices have gradually established the principle of proportionality, where the intensity of oversight corresponds to the level of research risk. For instance, the US FDA's Expanded Access mechanism and European Union's Compassionate Use framework provide critically ill patients with access to experimental therapies under strict ethical review and informed consent, offering an operational model that balances safety and innovation [278, 319]. Moreover, promoting mutual recognition in multi‐institutional ethical reviews can help avoid redundant evaluations, enhance transparency, and prevent ethical inconsistencies through public accountability mechanisms.

As digital technologies and AI become increasingly integrated into the field of transplantation, data governance has emerged as a central issue in next‐generation ethical compliance. The security, representativeness, and transparency of data during collection, sharing, and reuse directly influence the fairness of the algorithmic predictions and allocation decisions. Several international standards have established principles of data minimization, purpose limitation, and traceability; however, translating these principles into concrete operational norms for cross‐border organ transplantation data‐sharing networks remains a significant challenge. Recently, ethically trustworthy AI frameworks have been endorsed by multiple national regulatory authorities, emphasizing model bias detection, interpretability, and independent auditing [318]. Establishing dynamic regulatory mechanisms is essential at the practical level. Whenever algorithmic models are updated, their fairness and potential risks must be reassessed simultaneously. Moreover, the public has the right to understand how these algorithms influence organ allocation. The concept of explainable fairness is increasingly becoming a guiding principle in policy formulation and ethical oversight.

In summary, the evolution of organ transplantation ethics is not merely a chronicle of medical technological advancement but a reflection of modern society's ongoing negotiation between scientific rationality and moral responsibility. From early debates over the definition of death and consent models to contemporary reflections on algorithmic fairness and transnational regulation, each breakthrough in transplant medicine was accompanied by a reshaping of the ethical framework. Although technological progress continues to expand the boundaries of human medical practice, it exposes structural tensions involving resource allocation, public trust, and institutional legitimacy. Future governance must seek equilibrium through multidimensional coordination—anchoring ethical reviews as an institutional foundation, strengthening data governance and AI oversight as technological safeguards, and fostering public participation and international collaboration as social pillars. Only through such an integration can organ transplantation evolve into a paradigm that harmonizes efficiency with equity and innovation with responsibility, embodying the true coevolution of medical progress and human ethics.

## 8 Conclusion and Prospects

Organ transplantation is at the intersection of surgical innovation, immunological insights, and bioengineering. Over the past 50 years, it has transformed from a last‐resort therapy into a multidisciplinary enterprise that integrates genomics, immunomodulation, and regenerative medicine. Despite impressive advances in short‐term graft survival, long‐term outcomes remain constrained by chronic rejection and the persistent scarcity of donor organs. Future research and policy efforts should prioritize the following four strategies to bridge the gap between scientific innovation and routine clinical care.

*Standardization of Cellular Manufacturing and Potency Assays*: The transition of Treg cell and MSC therapies from early‐phase trials to standard care has been hindered by manufacturing variability. Future studies should prioritize the establishment of consensus protocols for cell expansion and reproducible potency assays. The validation of biomarker‐guided monitoring is essential to ensure reliable dosing and long‐term lineage stability in vivo.

*Evolution of Machine Perfusion into a Therapeutic Platform*: Feasibility studies have validated EVMP for preservation, and immediate clinical priority is now shifting toward therapeutic interventions. Research should focus on utilizing EVMP as a bioreactor for the targeted delivery of gene therapies, stem cell‐derived exosomes, and metabolic modulators to repair marginal grafts before implantation, thereby actively expanding the usable donor pool.

*Integration of Interpretable AI with Multiomics*: To maximize the impact of AI, the field must move beyond “black box” algorithms. Priority should be placed on developing interpretable predictive models that integrate clinical registries with high‐dimensional multiomics and imaging data. This requires the creation of multicenter, standardized data governance frameworks to prevent bias and ensure that AI tools can reliably guide precision immunosuppression.

*Regulatory Harmonization for Xenotransplantation*: As gene‐edited xenografts approach clinical viability, regulatory and ethical governance are the primary medical hurdles. Policy frameworks must be established to mandate rigorous lifelong pathogen surveillance and transparent ethical oversight. This is critical for mitigating infection risks and securing public trust in the safe introduction of animal‐derived organs into human recipients.

Looking forward, the next decade will likely witness a redefinition of transplantation as a discipline, not merely as a surgical replacement of failing organs but also as a platform for organ regeneration, tolerance, and bioengineered integration. Achieving this vision requires a sustained interdisciplinary collaboration across the fields of immunology, bioengineering, data science, and ethics. If realized, the evolution from “transplantation” to “regeneration” may not only extend graft longevity, but also transform organ replacement into a curative, self‐sustaining biological restoration process.

## Author Contributions

Xinqiang Li and Jinzhen Cai contributed to the research design. Xinqiang Li and Ruidong Ding contributed to the data management and wrote the manuscript. All authors contributed to the article and approved the submitted version.

## Ethics Statement

The authors have nothing to report.

## Conflicts of Interest

The authors declare no conflicts of interest.

## Funding

This work was supported by the National Natural Science Foundation of China (No. 82370666) and the Science Foundation of Shandong Province (No. ZR2022MH292).

## Data Availability

The authors have nothing to report.
